# Sex Differences in Running Performance Among 8‐and‐Under and 9–10‐Year‐Old Children in US Regional Track Championships

**DOI:** 10.1111/sms.70251

**Published:** 2026-03-08

**Authors:** Gregory A. Brown, Brandon S. Shaw, Ina Shaw

**Affiliations:** ^1^ Physical Activity and Wellness Laboratory, Department of Kinesiology and Sport Sciences University of Nebraska at Kearney Kearney Nebraska USA; ^2^ School of Sport, Rehabilitation and Exercise Sciences University of Essex Colchester UK; ^3^ Division of Public Health University of the Free State Bloemfontein South Africa

**Keywords:** prepubertal children, running performance, sex differences, track and field, youth athletics

## Abstract

Sex differences in competitive running performance are well documented in adults but understudied in prepubertal children. Prior studies have often focused on elite performers or record holders, potentially neglecting trends across the full competitive field, and some have reported minimal sex‐based differences before age 11, leaving the existence of prepubertal performance differences unclear. This study examined whether sex‐based differences in running performance are present among all competitors in the 8‐and‐under and 9–10‐year‐old age groups at USA Track & Field (USATF) Regional Junior Olympic Championships. All official race times from the 100 m, 200 m, 400 m, 800 m, and 1500 m events were collected from all 15 USATF Regional Championships held in 2022–2024. Preliminary and final race times were compared by sex using two‐way ANOVA (*p* < 0.05), and performance differences were further evaluated across the 95th, 90th, 75th, 50th, 25th, and 10th percentiles. In the 8‐and‐under group, males (*n* = 2696 race times) ran 3.8%–5.9% faster than females (*n* = 2710) across all distances (*p* < 0.001; Hedges' *g* = 0.39–0.63). In the 9–10‐year‐old group, males (*n* = 3265) ran 3.4%–5.5% faster than females (*n* = 3182) across all distances (*p* < 0.001; Hedges' *g* = 0.31–0.52). The fastest individual males outperformed the fastest individual females by 1.8%–11.6% across events and age groups. Across percentiles, males ran on average 4.4% ± 1.2% faster than females. These findings demonstrate that sex‐based differences in running performance are evident by ages 8–10 years and extend across the full spectrum of competitors, not solely elite youth athletes.

## 1 Introduction

According to position statements from the American College of Sports Medicine and the Endocrine Society, sex refers to a set of biological characteristics, including chromosomes, hormone concentrations, and reproductive anatomy, that distinguish males from females [1, 2]. In contrast, gender is a multidimensional construct shaped by cultural, social, and psychological influences, encompassing roles, behaviors, and identities commonly associated with masculinity or femininity. The longstanding practice of separating athletes by sex in competitive sport has increasingly been questioned as discussions surrounding sex and gender intersect with concerns related to performance advantages, competitive fairness, and inclusion in sport [3, 4]. In the present manuscript, the terms *male* and *female* are used to denote biological sex classification, consistent with the definitions above and with terminology used in recent research on sex‐based differences in sport.

Despite this evolving discourse, sex remains a well‐established determinant of athletic performance. In adults, males typically outperform females by approximately 10%–40% in activities dependent on cardiorespiratory endurance, muscular strength, speed, and power [2, 5, 6]. These differences are largely attributed to anatomical and physiological changes that occur during puberty, particularly the marked increase in testosterone secretion in males causing increases in muscle mass and hemoglobin concentrations [7, 8]. While some prior statements have characterized prepubertal sex‐based performance differences as minimal [2] or nonexistent [8], and some research has suggested that differences do not emerge before approximately 11 years of age [9], a growing body of evidence indicates that measurable sex‐based differences in physical performance are present at 6–10 years of age [7, 10, 11, 12, 13, 14, 15, 16] (i.e., before the age typically associated with male puberty). Although the onset of puberty varies between individuals and may occur earlier in some children, particularly girls, most athletes within the 8‐and‐under and 9–10‐year‐old age groups fall below the typical age range for pubertal hormonal divergence [7, 17, 18]. Although these differences are smaller than those observed after puberty, they are nonetheless consistent, ranging from approximately 1%–2% in swimming [7, 13, 15], 3%–8% in running and jumping events [7, 10, 11, 12, 14, 15], and from 6% to over 30% in throwing performance [12].

Notably, much of the existing research examining prepubertal sex‐based performance differences has focused on elite youth athletes, such as top finishers at national championships or those ranked among the highest performers [7, 10, 11, 12, 13, 14, 15, 16]. While informative for understanding peak performance, this approach limits generalizability to the broader population of youth competitors. Christensen and Griffiths [14] examined performance among all 6–12‐year‐old participants in a 1600 m race and reported that males were faster than females across all ages. However, there is a dearth of similar analyses including all competitors across a wider range of running distances.

Restricting analyses to elite or top‐ranked youth athletes may obscure whether observed sex‐based differences reflect population‐wide patterns or are confined to the highest‐performing individuals. Analyses that include all competitors allow assessment of performance differences across the full distribution of ability, reducing selection bias and improving generalizability. Evaluating sex‐based differences across multiple percentiles further clarifies whether performance disparities are consistent throughout the population or emerge primarily at the upper extremes of performance. Such an approach enhances ecological validity by reflecting real‐world competitive environments and provides a more comprehensive understanding of prepubertal performance differences relevant to coaches, practitioners, and sport governing bodies. Because running, particularly sprinting, is a foundational component of performance in many other sports [6], evaluation of sex‐based differences in running may also offer insight into broader sex‐based performance differences across athletic domains.

Therefore, the purpose of the present study was to evaluate sex‐based differences in running performance among prepubertal males and females across distances ranging from 100 to 1500 m in regional track competitions throughout the United States. By including all eligible competitors across multiple events and competitions spanning the country, this study aims to provide a comprehensive assessment of performance patterns in this age group. These findings may enhance understanding of prepubertal sex‐based differences in athletic performance and inform evidence‐based approaches to training, talent identification, and youth sport development in track and field and other running‐based sports.

## 2 Methods

### 2.1 Subjects

USA Track & Field (USATF) divides the United States into 15 youth regions, each of which typically hosts an annual outdoor Regional Junior Olympic Track & Field Championship [19]. The location of these championships rotates among USATF state‐level associations within each region. Athletes generally qualify for regional competition by placing among the top eight finishers in their respective state‐level USATF association championships; however, qualification criteria may be adjusted at the discretion of regional coordinators to accommodate local circumstances [20].

Performance data were obtained for all competitors in the 100 m, 200 m, 400 m, 800 m, and 1500 m events within the 8‐and‐under and 9–10‐year‐old age groups across all 15 regions during the 2022, 2023, and 2024 Regional Junior Olympic Championships. These age groups were selected because they represent the youngest divisions in USATF competition and include athletes below the typical age of onset of male puberty (11.5 years) [2, 17] and associated increase in testosterone secretion [7, 18].

A priori sample size calculations were based on previously reported 100 m sex‐based differences among 8‐and‐under athletes (males: 14.97 ± 0.97 s; females: 15.58 ± 0.96 s) [11]. Using G*Power v3.1 [21], a minimum of 40 participants per sex was required to detect a significant difference with 80% power at *α* = 0.05.

Data were collected from publicly available, official meet results posted on Athletic.net. Since the study used publicly accessible, non‐identifiable data, it did not require institutional ethical review in accordance with 45 CFR 46.102 and the principles outlined in the Declaration of Helsinki and as affirmed by the Institutional Review Board at the University of Nebraska at Kearney.

### 2.2 Procedures

All official finishing times were extracted from publicly available meet results. Data were downloaded as region‐ and year‐specific datasets from Athletic.net between July 1, 2024, and February 28, 2025.

Data entry was performed by a single researcher (G.A.B.) using Microsoft Excel 365 (Microsoft Corp., Redmond, WA). The data were cross‐verified for transcription accuracy by two office associates unaffiliated with the project, and corrections were made where necessary. For the 100 m, 200 m, and 400 m events, times were listed as either preliminary or final. For the 800 m and 1500 m events, only final times were available, as these events were conducted without preliminary heats.

The USATF Regional Junior Olympic Championships were selected for analysis because they provide a geographically broad, age‐stratified sampling of youth athletes, conducted under standardized eligibility and timing regulations. One exception was Region 3, where the 2024 championship was canceled; all other regions held competitions in each of the 3 years.

### 2.3 Statistical Analyses

All data are presented as mean ± standard deviation unless otherwise noted. Times for 400 m, 800 m, and 1500 m were recorded in minutes:seconds (min:s) and were converted to seconds (s) for statistical analysis and uniform presentation. For the 100 m, 200 m, and 400 m events, two‐way ANOVAs (factors: sex × time type [all times combined, preliminary, or final]) were used to evaluate performance differences within each age group. For the 800 m and 1500 m events, where only final times were available and the data failed the Shapiro–Wilk normality tests (*p* < 0.05), comparisons between males and females were made using the Mann–Whitney U test (SigmaStat 4.0, Systat Software, San Jose, CA). Statistical significance was set at *p* < 0.05.

Effect sizes were calculated using Hedges' *g* to account for unequal sample sizes between groups. Percent differences in performance between sexes were calculated following the formula described by Handelsman [7], which represents the percent male advantage:

$$\frac{\text{Female time}-\text{male time}}{\text{male time}}\times 100$$

To evaluate performance distribution, percentiles (10th, 25th, 50th, 75th, 90th, and 95th) were computed for each sex and age group using the PERCENTILE function in Microsoft Excel. Data for each event were sorted by performance, and percentiles were calculated based on all valid finishing times.

## 3 Results

When data across all age groups and race distances were pooled, average finishing times for males were 4.4% ± 0.9% faster than for females. The fastest male times were faster than the fastest female times by 5.7% ± 3.3%. Across all events and age groups, males demonstrated 3%–8% faster times at every percentile examined (10th–95th; Table 1), and both average and fastest‐individual race times were consistently lower for males.

**TABLE 1:** Percentile race times for males and females in the 100 m, 200 m, 400 m, 800 m, and 1500 m events for the 8‐and‐under and 9–10‐year‐old age groups, based on data from the USA Track & Field Regional Junior Olympic Championships (2022–2024).

Percentile	8‐and under		9–10‐year‐old	
	Male (s)	Female (s)	Male (s)	Female (s)
100 m				
95th	14.58	15.21	13.51	13.99
90th	14.80	15.50	13.77	14.23
75th	15.43	16.04	14.19	14.68
50th	16.17	16.89	14.71	15.27
25th	17.12	17.95	15.48	16.01
10th	18.14	18.99	16.36	16.97
200 m				
95th	30.35	31.73	27.71	28.60
90th	30.93	32.48	28.25	29.23
75th	32.41	33.70	29.21	30.25
50th	34.04	35.43	30.66	31.75
25th	36.32	37.73	32.40	33.60
10th	38.82	40.38	34.59	35.52
400 m				
95th	69.73	73.57	63.41	65.20
90th	71.90	74.53	64.90	66.78
75th	75.10	78.31	67.27	69.75
50th	79.67	84.08	71.06	74.08
25th	84.69	91.16	76.73	78.93
10th	90.81	97.90	83.53	85.06
800 m				
95th	166.40	173.28	152.24	157.41
90th	169.68	178.71	154.18	162.15
75th	176.22	186.59	161.17	168.32
50th	187.61	197.78	169.52	177.16
25th	199.83	211.61	180.69	188.74
10th	214.49	228.69	192.59	203.51
1500 m				
95th	336.81	353.16	311.43	320.53
90th	343.89	361.88	318.58	328.72
75th	356.55	377.56	328.01	344.20
50th	382.74	397.42	344.68	363.66
25th	412.70	426.85	368.81	387.27
10th	445.18	465.88	392.65	416.10

### 3.1 100 m (8‐and‐Under Age Group)

**FIGURE 1:**
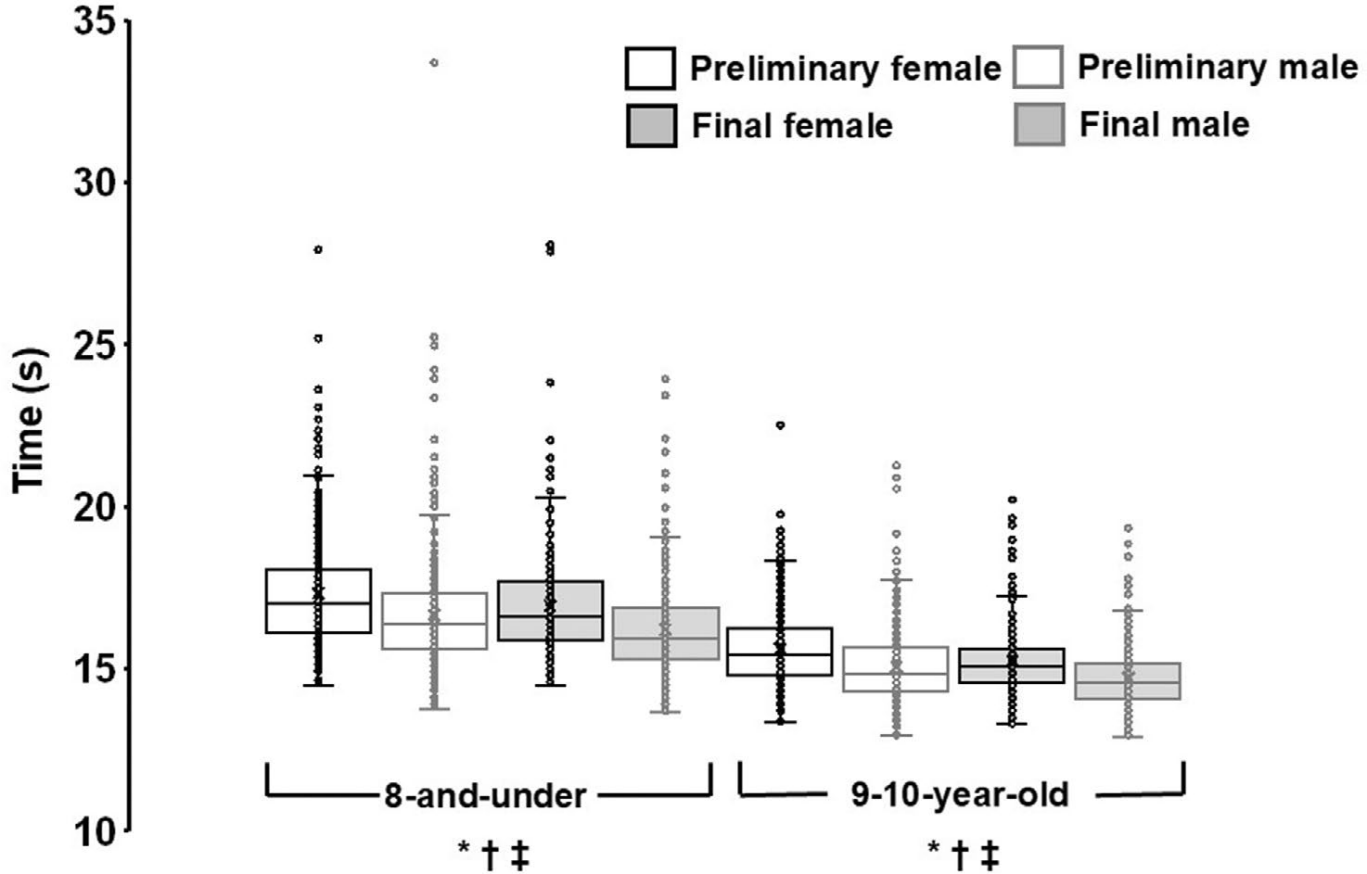
Box‐and‐whisker plots of 100 m race times (s) from all USA Track & Field Regional Junior Olympic Championships, 2022–2024. Boxes represent the interquartile range (25th–75th percentiles), with the medians shown as solid horizontal lines; whiskers extend to 1.5 × IQR. Means are denoted by “X.” *p < 0.001 for preliminary versus final race times (main effect). ^†^ *p* < 0.001 for male versus female preliminary times. ^‡^ *p* < 0.001 for male versus female final times.

Preliminary times were available from 28 meets for males and 32 meets for females (Table 2). Final‐only times were recorded at 16 meets for males and 12 meets for females. Final times were significantly faster than preliminary times for both sexes (*p* < 0.001). Across all performances, males ran 4.4% faster than females (*p* < 0.001; Figure 1). This difference was observed in both preliminary and final races (all *p* < 0.001). The fastest male was 5.7% faster than the fastest female, and 24 males recorded times faster than the fastest female.

**TABLE 2:** Race times for males and females in the 100 m, 200 m, and 400 m events in the 8‐and‐under and 9–10‐year‐old age groups, based on data from the USA Track & Field Regional Junior Olympic Championships (2022–2024).

Age group	Round	Male			Female			Sex difference (*p*)	Effect size (male versus female; Hedges' *g*)	Within‐sex preliminary versus final (*p*)
		*n*	Mean ± SD (s)	Range (all times; s)	*n*	Mean ± SD (s)	Range (all times; s)			
Distance: 100 m										
8‐and‐under	All times	798	16.46 ± 1.68	13.68–33.70	866	17.18 ± 1.68	14.46–28.08	< 0.001	0.429	
	Preliminary	455	16.66 ± 1.79		506	17.34 ± 1.69		< 0.001	0.391	M: < 0.001 F: < 0.001
	Final	343	16.21 ± 1.49		360	16.95 ± 1.64		< 0.001	0.454	
9–10‐year‐old	All times	913	14.93 ± 1.10	12.89–22.28	919	15.47 ± 1.18	13.30–22.52	< 0.001	0.473	
	Preliminary	546	15.08 ± 1.14		552	15.62 ± 1.20		< 0.001	0.470	M: < 0.001 F: < 0.001
	Final	367	14.72 ± 1.02		367	15.25 ± 1.13		< 0.001	0.492	
Distance: 200 m										
8‐and‐under	All times	775	34.69 ± 3.40	28.68–54.33	790	36.22 ± 3.84	29.53–66.42	< 0.001	0.424	
	Preliminary	430	35.20 ± 3.48		439	36.61 ± 3.79		< 0.001	0.387	M: < 0.001 F: < 0.001
	Final	345	34.03 ± 3.18		351	35.73 ± 3.85		< 0.001	0.481	
9–10‐year‐old	All times	866	31.07 ± 2.64	25.53–45.04	833	32.19 ± 2.85	26.78–55.51	< 0.001	0.408	
	Preliminary	486	31.49 ± 2.99		467	32.64 ± 3.07		< 0.001	0.380	M: 0.010 F: 0.005
	Final	380	30.80 ± 2.56		366	31.84 ± 2.82		< 0.001	0.387	
Distance: 400 m										
8‐and‐under	All times	577	80.88 ± 8.39	62.74–122.72	592	85.63 ± 9.58	69.43–130.51	< 0.001	0.527	
	Preliminary	228	80.55 ± 7.37		248	86.27 ± 10.40		< 0.001	0.630	M: 0.477 F: 0.143
	Final	349	81.10 ± 9.00		344	85.17 ± 8.92		< 0.001	0.454	
9–10‐year‐old	All times	655	72.83 ± 7.61	57.50–129.22	681	75.29 ± 8.08	59.53–130.38	< 0.001	0.313	
	Preliminary	250	72.56 ± 7.75		267	75.06 ± 7.70		< 0.001	0.324	M: 0.486 F: 0.610
	Final	405	73.00 ± 7.53		414	75.45 ± 8.32		< 0.001	0.309	

### 3.2 100 m (9–10‐Year‐Old Age Group)

Preliminary times were available from 34 meets for males and 32 meets for females (Table 2). Final‐only times were recorded at 10 meets for males and 12 meets for females. Final times were significantly faster than preliminary times for both sexes (*p* < 0.001). Across all performances, males ran 3.6% faster than females (*p* < 0.001; Figure 1). This difference was observed in both preliminary and final races (all *p* < 0.001). The fastest male recorded a time that was 3.2% faster than the fastest female, and 23 males recorded times faster than the fastest female.

### 3.3 200 m (8‐and‐Under Age Group)

**FIGURE 2:**
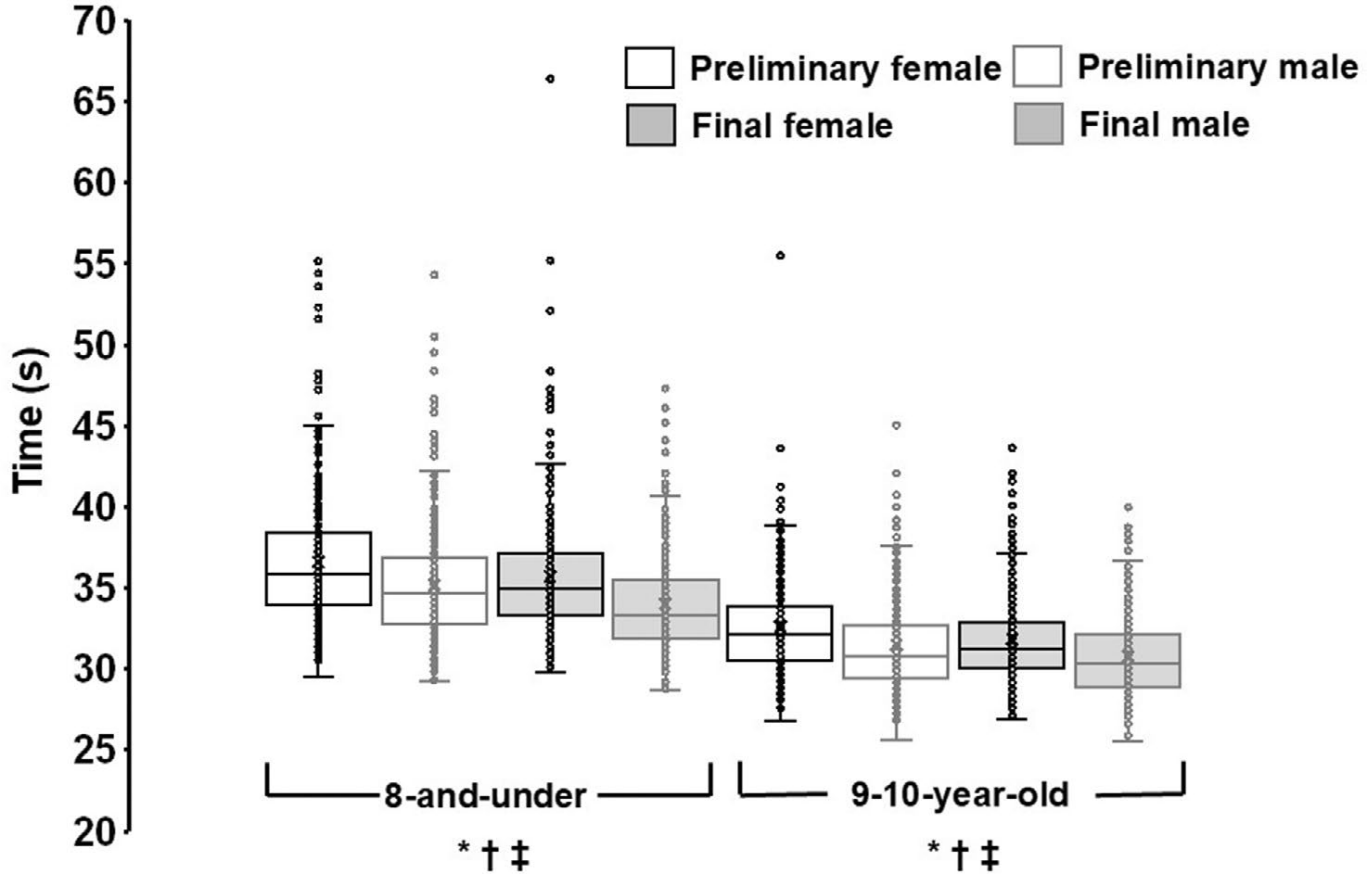
Box‐and‐whisker plots of 200 m race times (s) from all USA Track & Field Regional Junior Olympic Championships, 2022–2024. Boxes represent the interquartile range (25th–75th percentiles), with the medians shown as solid horizontal lines; whiskers extend to 1.5 × IQR. Means are denoted by “X.” *p < 0.001 for preliminary versus final race times (main effect). ^†^ *p* < 0.001 for male versus female preliminary times. ^‡^ *p* < 0.001 for male versus female final times.

Preliminary times were available from 25 meets for males and 27 meets for females (Table 2). Final‐only times were recorded at 19 meets for males and 17 meets for females. Final times were significantly faster than preliminary times for both sexes (*p* < 0.001). Across all performances, males ran 4.4% faster than females (*p* < 0.001; Figure 2). This difference was observed in both preliminary and final races (all *p* < 0.001). The fastest male was 3.0% faster than the fastest female, and 12 males recorded times faster than the fastest female.

### 3.4 200 m (9–10‐Year‐Old Age Group)

Preliminary times were available from 29 meets for males and 27 meets for females (Table 2). Final‐only times were recorded at 15 meets for males and 17 meets for females. Final times were significantly faster than preliminary times for both males (*p* = 0.010) and females (*p* = 0.005). Across all performances, males ran 3.6% faster than females (*p* < 0.001; Figure 2). This difference was observed in both preliminary and final races (all *p* < 0.001). The fastest male recorded a time that was 4.9% faster than the fastest female, and six males recorded times faster than the fastest female.

### 3.5 400 m (8‐and‐Under Age Group)

**FIGURE 3:**
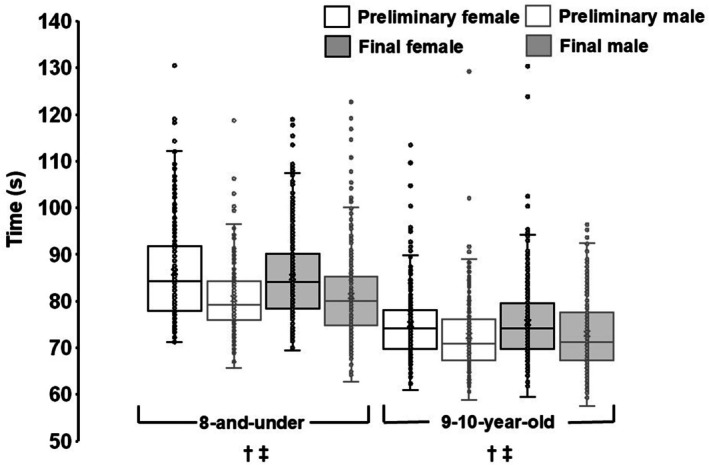
Box‐and‐whisker plots of 400 m race times (s) from all USA Track & Field Regional Junior Olympic Championships, 2022–2024. Boxes represent the interquartile range (25th–75th percentiles), with the medians shown as solid horizontal lines; whiskers extend to 1.5 × IQR. Means are denoted by “X.” There was no significant difference between preliminary and final race times. ^†^ *p* < 0.001 for male versus female preliminary times. ^‡^ *p* < 0.001 for male versus female final race times.

Preliminary times were available from 15 meets for males and 17 meets for females (Table 2). Final‐only times were recorded at 29 meets for males and 26 meets for females. There were no significant differences between preliminary and final times for either sex. Across all performances, males ran 5.9% faster than females (*p* < 0.001; Figure 3). Similar differences were observed when preliminary and final races were analyzed separately (all *p* < 0.001). The fastest male ran 10.7% faster than the fastest female, and 23 males recorded times faster than the fastest female.

### 3.6 400 m (9–10‐Year‐Old Age Group)

Preliminary times were available from 16 meets for males and 16 meets for females (Table 2). Final‐only times were recorded at 28 meets for males and 28 meets for females. There were no significant differences between preliminary and final times for either sex. Across all performances, males ran 3.4% faster than females (*p* < 0.001; Figure 3). This difference was observed in both preliminary and final races (all *p* < 0.001). The fastest male recorded a time that was 3.5% lower than the fastest female, and three males recorded times faster than the fastest female.

### 3.7 800 m (8‐and‐Under Age Group)

**FIGURE 4:**
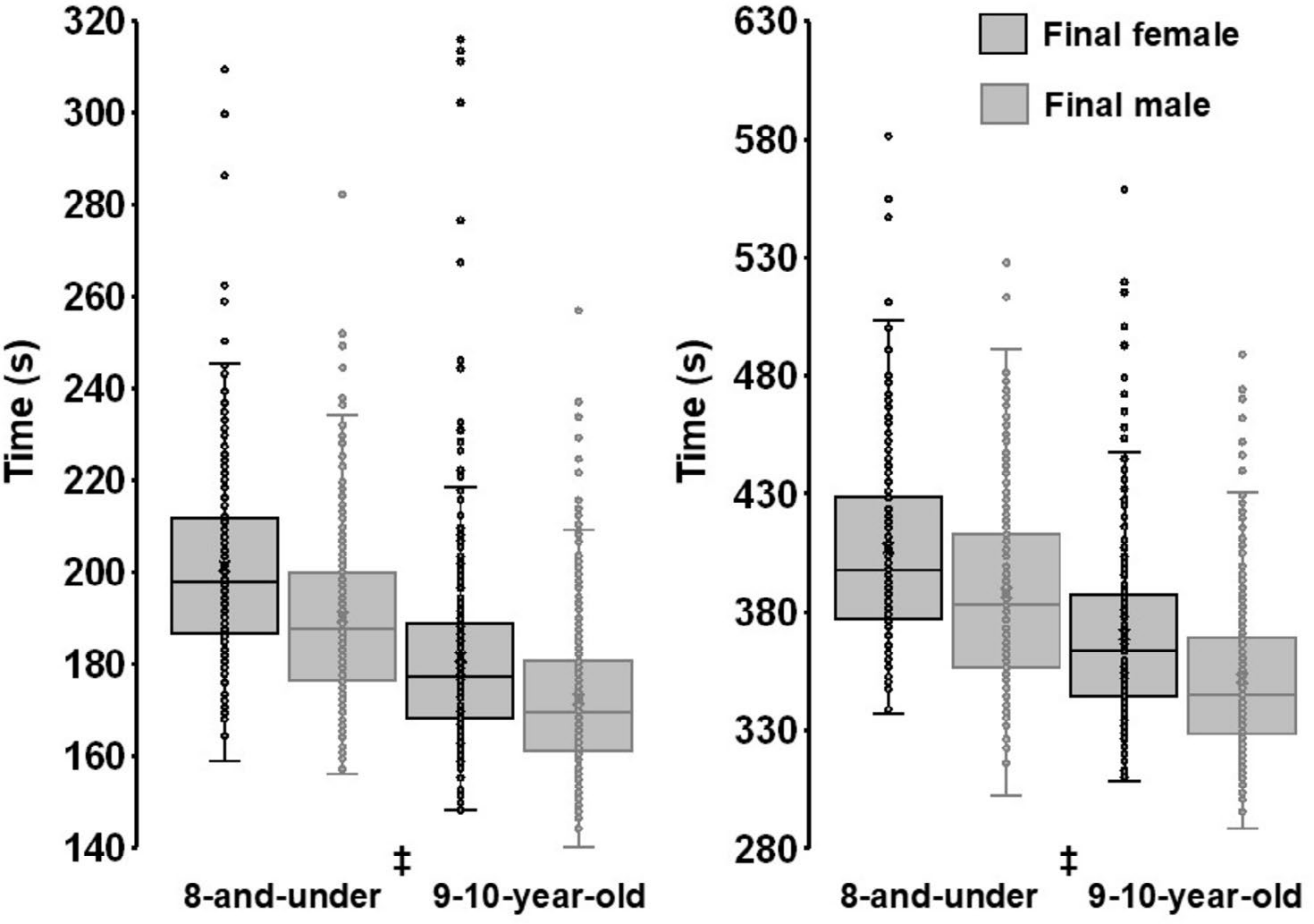
Box‐and‐whisker plots of 800 m (left panel) and 1500 m (right panel) race times (s) from all USA Track & Field Regional Junior Olympic Championships, 2022–2024. Boxes represent the interquartile range (25th—75th percentiles), with medians shown as solid horizontal lines; whiskers extend 1.5 × IQR. Means are denoted by “X.” ^‡^ *p* < 0.001 male versus female race time.

The 800 m was conducted as a finals‐only event (Table 3). Males ran 5.4% faster than females (Mann–Whitney U test, *p* < 0.001; Figure 4). The fastest male was 1.8% faster than the fastest female, and three males recorded times faster than the fastest female.

**TABLE 3:** 800 m and 1500 m race times for males and females in the 8‐and‐under and 9–10‐year‐old age groups, based on data from the USA Track & Field Regional Junior Olympic Championships (2022–2024).

Age group	Round	Males			Females			Sex difference		
		*n*	Median (s)	Range (s)	*n*	Median (s)	Range (s)	(*p*)	(*U*)	Effect size (Hedges' *g*)
Distance: 800 m										
8‐and‐under	Finals	314	187.61	155.92–282.17	280	197.78	158.66–309.46	< 0.001	29 589	0.552
9–10‐year‐old	Finals	468	169.52	140.06–256.87	430	177.16	148.21–315.92	< 0.001	72 495	0.470
Distance: 1500 m										
8‐and‐under	Finals	232	382.74	301.79–529.29	183	397.42	336.80–581.39	< 0.001	15 420	0.464
9–10‐year‐old	Finals	387	344.68	288.14–488.96	320	363.66	307.79–558.91	< 0.001	42 603	0.522

*Note:* 800 m and 1500 m events were conducted as finals‐only races.

### 3.8 800 m (9–10‐Year‐Old Age Group)

The 800 m was conducted as a finals‐only event (Table 3). Males ran 4.5% faster than females (Mann–Whitney U test, *p* < 0.001; Figure 4). The fastest male recorded a time 5.8% lower than the fastest female, and nine males recorded times faster than the fastest female.

### 3.9 1500 m (8‐And‐Under Age Group)

The 1500 m was conducted as a finals‐only event (Table 3). Males ran 3.8% faster than females (Mann–Whitney *U* test, *p* < 0.001; Figure 4). The fastest male was 11.6% faster than the fastest female, and 12 males recorded times faster than the fastest female.

### 3.10 1500 m (9–10‐Year‐Old Age Group)

The 1500 m was conducted as a finals‐only event (Table 3). Males ran 5.5% faster than females (Mann–Whitney *U* test, *p* < 0.001; Figure 4). The fastest male recorded a time 6.8% faster than the fastest female, and 14 males recorded times faster than the fastest female.

## 4 Discussion

This study evaluated all finishing times in both the 8‐and‐under and 9–10‐year‐old age groups from the 100 m, 200 m, 400 m, 800 m, and 1500 m events contested at the USATF Regional Junior Olympic Championships between 2022 and 2024. Across all distances, males consistently outperformed females, demonstrating faster mean performances as well as faster fastest‐individual times. These sex‐based differences were associated with small‐to‐moderate effect sizes, indicating that although the absolute differences were modest, they were consistent and meaningful—particularly in the context of competition, where race outcomes are often determined by narrow margins. These findings align with prior studies reporting male performance advantages in running events among prepubertal children [7, 10, 11, 14, 15], and extend the literature by examining a large, representative sample that includes the full distribution of competitors in real‐world youth competitions.

Faster race times in males were evident across all distances, with no clear pattern of increasing or decreasing magnitude based on race distance. Average sex differences ranged from 3.4% (400 m, 9–10‐year‐olds) to 5.9% (400 m, 8‐and‐under), remaining within that relatively narrow range across sprint and middle‐distance events. Differences between the fastest individual male and female performances showed greater variability, ranging from 1.8% (800 m, 8‐and‐under) to 11.6% (1500 m, 8‐and‐under). However, these larger values reflect single top performances rather than overall distributional trends. Collectively, the findings suggest that prepubertal sex‐based differences in running are not confined to either sprint or middle‐distance events but are broadly evident across race types. The absence of a distance‐dependent pattern suggests that early sex‐based differences may reflect general performance capacities rather than mechanisms specific to anaerobic or aerobic demands. The present findings provide evidence inconsistent with reports suggesting that prepubertal sex differences are minimal or absent. In the present study, males ran approximately 3%–6% faster than females across all distances from 100 m to 1500 m, and this magnitude was consistent across percentiles and age groups. These findings contrast with reports suggesting that sex‐based differences prior to puberty are minimal or absent [2, 8, 9], while some prior reports have suggested that meaningful sex differences do not emerge before approximately 11 years of age [9]. In contrast, a growing body of empirical evidence indicates that males run approximately 3%–8% faster than age‐matched females, even prior to the age typically associated with the onset of puberty [7, 10, 11, 14, 15]. Most previous investigations in this area have focused on high‐performing youth athletes [7, 10, 11, 15], with only one study to date examining all competitors aged 6–12 years in a single running distance (1600 m) irrespective of performance level [14]. By including all participants across multiple distances ranging from 100 m to 1500 m, the present study provides stronger evidence that male performance advantages are evident across the full spectrum of ability in early childhood.

Prepubertal sex‐based differences in running performance are also well documented in standardized physical fitness assessments. For example, data from the 1976 AAPHER Youth Fitness Test showed that males aged 10 years and under outperformed females in the shuttle run, 50‐yard dash, and 600‐yard run [22], findings that have been confirmed in more recent fitness testing studies [23, 24, 25, 26, 27]. Importantly, the participants in the present regional championship meets qualified based on time or placement criteria and therefore represent a competitively engaged, sub‐elite to elite cohort rather than a general population sample. Although these populations differ in selection and performance level, the observation of similar sex‐based performance differences across both unselected school‐based samples and competitively selected youth athletes suggests that male advantages are not confined to either the general population or only the highest‐performing individuals. While superior fitness test performance does not always translate directly to competitive success, the present findings demonstrate that male advantages observed in testing environments are also evident in real‐world competitive racing. Together, these results strengthen evidence that prepubertal sex‐based performance differences are present across both testing and competition contexts. Although the ~3%–6% average faster race times observed in males in the present data is smaller than the ~10%–15% difference typically reported in adults [2, 6], even modest differences can meaningfully influence competitive outcomes. Although boys and girls typically compete in sex‐segregated events, even modest performance differences are relevant in contexts where sex categories are debated or where training standards, talent identification benchmarks, or qualification criteria are compared across sexes. In track events in adults, margins separating finalists or podium positions are often less than 1% [28, 29, 30]. From this perspective, relatively small but consistent performance advantages may have disproportionate implications for competitive placement. Because sprinting ability contributes to performance in numerous field and court sports [6], even modest sex‐based differences in running speed may have practical implications across a wide range of youth athletic contexts.

The present study did not directly assess the mechanisms underlying the observed performance differences. Biological factors likely contribute to the male advantages observed. Previous reports indicate that prepubertal males possess approximately 10% greater lean body mass than females [31, 32], which may enhance running performance. Additional factors include modest advantages in cardiovascular and pulmonary structure, as well as pelvic morphology [32], with males exhibiting narrower ischial and acetabular widths that may promote more efficient running mechanics [33], which could contribute further. Although circulating testosterone and hemoglobin concentrations do not differ substantially between prepubertal males and females [6, 20, 31, 34], these morphological and compositional differences may reflect other biological influences established early in development [6, 34].

Behavioral and sociocultural factors may further amplify these differences. Boys may be more frequently encouraged to participate in running‐intensive activities or competitive sport during early childhood, whereas girls may be disproportionately represented in activities emphasizing flexibility, coordination, or aesthetic performance. Such environmental influences could contribute to sex‐based differences in training exposure and motor skill development [35, 36, 37]. However, previous research has indicated that activity differences alone do not fully explain sex‐based disparities in physiological variables such as VO_2_max or body composition [38, 39].

Notably, participation numbers in the present dataset were broadly similar between males and females in the 100 m, 200 m, and 400 m events, suggesting that gross differences in entry volume alone are unlikely to account for the observed performance disparities. This aligns with Christensen et al. [14], who reported that participation rates in a 1600 m run among children aged 6–12 years did not explain sex‐based differences in race times. Nevertheless, similar participation counts do not necessarily indicate equivalent training volume, sport specialization patterns, or intensity of practice, and these factors were not assessed in the present study. Accordingly, the relative contributions of biological and sociocultural influences cannot be determined from the current data. Beyond structural and participation‐related factors, race execution strategies may also influence observed performance differences, particularly in middle‐distance events.

It is also possible that pacing strategies contribute to observed differences in middle‐distance events. In adults, sex‐based differences in pacing have been reported, with women often exhibiting more even pacing patterns than men [40]. Whether similar behavioral pacing differences exist in prepubertal children is unknown. Unfortunately, the records used for evaluation in this project only include finishing times and not lap times. Future research incorporating split times could help determine whether performance gaps in longer youth races reflect physiological capacity, pacing strategy, or both.

One limitation of the present analysis is that athletes were classified by chronological age, and pubertal status was not directly assessed. However, endocrinological evidence supports the assumption that the age groups examined represent predominantly prepubertal populations. Both Handelsman [7] and Senefeld et al. [20] report that circulating testosterone concentrations do not differ meaningfully between males and females prior to approximately 11 years of age. In addition, precocious puberty in males is rare relative to females and is typically defined by pubertal onset before 9–10 years of age [19], making it unlikely that early pubertal maturation accounts for the population‐wide differences observed. Importantly, sex‐based running performance differences of similar magnitude have been reported in younger children well below the age range examined here. Among 6‐year‐olds, Christensen and Griffiths [14] observed male advantages in 1600 m running comparable to those in the present study. Tambalis et al. [25] likewise reported approximately 3% faster performance in 6‐year‐old males during a 50 m shuttle run. Collectively, these findings indicate that the observed differences are unlikely to be explained by pubertal maturation.

Future research should aim to integrate physiological, biomechanical, and behavioral assessments with competitive performance data to better explain the mechanisms underlying early sex‐based performance differences. Longitudinal designs tracking children's performance across pre‐, peri‐, and post‐puberty would be particularly valuable in clarifying when and how the sex‐based differences in athletic performance increase. Additionally, analyses incorporating training exposure, sport participation patterns, and pacing metrics could help disentangle biological from environmental contributors.

In conclusion, sex‐based differences in athletic performance among prepubertal children have sometimes been characterized as minimal or absent; the present findings provide evidence inconsistent with that interpretation. While individual females occasionally outperform individual males, males in both the 8‐and‐under and 9–10‐year‐old age groups ran faster on average and achieved faster fastest individual times across all distances from 100 m to 1500 m. Although biological mechanisms underlying these differences cannot be directly established in the absence of individual‐level physiological data, the consistent ~3%–8% performance advantage observed across events and ability levels provides strong evidence of meaningful sex‐based differences in running performance before puberty. These findings contribute to a more complete understanding of youth athletic performance and have relevance for evidence‐based decision‐making in youth sport development and sex‐segregated competition.

## 5 Perspective

Sex‐based differences in athletic performance are well established in adult populations and have recently received increasing attention in children. Previous research has documented modest prepubertal male advantages in elite cohorts, yet questions have remained regarding their generalizability across the full competitive field. By examining comprehensive regional championship data across sprint and middle‐distance running, the present study contributes to a broader understanding of early sex‐based performance differences in organized sport settings. Within sports medicine, these findings may inform discussions and future research related to youth athlete development, benchmarking standards, and longitudinal performance tracking. Importantly, recognizing that measurable sex‐based differences may be present prior to puberty does not resolve questions regarding causation; rather, it underscores the need for mechanistic and longitudinal research integrating physiology, biomechanics, and training exposure. Future research should also examine whether similar sex‐based differences are evident across other sports, including team sports and skill‐based disciplines, to clarify the generalizability of early sex‐based differences beyond track and field. As sports medicine continues to refine evidence‐based approaches to athlete development and competition policy, clearer characterization of performance patterns across childhood will remain essential.

## Funding

The authors have nothing to report.

## Ethics Statement

The authors have nothing to report.

## Consent

The authors have nothing to report.

## Conflicts of Interest

The authors declare no conflicts of interest.

## Data Availability

All data used in this study were obtained from a publicly accessible database (www.athletic.net).
